# Stanniocalcin1 (STC1) Inhibits Cell Proliferation and Invasion of Cervical Cancer Cells

**DOI:** 10.1371/journal.pone.0053989

**Published:** 2013-01-29

**Authors:** Fengjie Guo, Yalin Li, Jiajia Wang, Yandong Li, Yuehui Li, Guancheng Li

**Affiliations:** 1 Cancer Research Institute, Xiangya Medical School, Central South University, Changsha, China; 2 Key Laboratory of Carcinogenesis and Cancer Invasion, Ministry of Education, Changsha, China; 3 Key Laboratory of Carcinogenesis, Ministry of Health, Changsha, China; 4 Tianjin Key Laboratory of Lung Cancer Metastasis and Tumor Microenvironment, Tianjin Lung Cancer Institute, Tianjin Medical University General Hospital, Tianjin, China; Children’s Hospital Boston & Harvard Medical School, United States of America

## Abstract

STC1 is a glycoprotein hormone involved in calcium/phosphate (Pi) homeostasis. There is mounting evidence that STC1 is tightly associated with the development of cancer. But the function of STC1 in cancer is not fully understood. Here, we found that STC1 is down-regulated in Clinical tissues of cervical cancer compared to the adjacent normal cervical tissues (15 cases). Subsequently, the expression of STC1 was knocked down by RNA interference in cervical cancer CaSki cells and the low expression promoted cell growth, migration and invasion. We also found that STC1 overexpression inhibited cell proliferation and invasion of cervical cancer cells. Moreover, STC1 overexpression sensitized CaSki cells to drugs. Further, we showed that NF-κB p65 protein directly bound to STC1 promoter and activated the expression of STC1 in cervical cancer cells. Thus, these results provided evidence that STC1 inhibited cell proliferation and invasion through NF-κB p65 activation in cervical cancer.

## Introduction

Stanniocalcin1 (STC1) is a glycoprotein hormone involved in calcium/phosphate (Pi) homeostasis, which was originally discovered in a secretory hormone of the corpuscles of Stannius, an endocrine gland of bony fish [1]. The mammalian STC1 is broadly expressed in various tissues including heart, lung, liver, adrenal, kidney, ovary, prostate, colon and spleen [2]–[5]. STC1 plays diverse role in physiological and pathological processes, including pregnancy, lactation, angiogenesis, organogenesis, oxidative stress, cerebral ischemia, and apoptosis [6]–[10]. A human ortholog of fish STC1 was found by mRNA differential display of cancer related genes [11]. STC1 was originally cloned in a screen for cancer-related genes. Numerous lines of studies indicated that altered expression of STC1 may have a role in carcinogenesis and development. Increased STC1 gene expression has been found in hepatocellular, colorectal, acute leukemia, and medullary thyroid carcinomas, however, decreased expression of STC1 expression was found in breast and ovarian cancer cell lines [12]–[19]. But the relationship between the differentially expression of STC1 in tumor compared to normal tissue and its biological function needs further investigation. Some study indicated that STC1 expression was involved in the formation of tumor vasculature, and STC1 can induce adaptive responsive to hypoxia by HIF regulation in human cancer cells [20], [21]. It is reported that histone deacetylase inhibitor-induced cellular apoptosis involves STC1 activation [22].

Despite of enhanced knowledge of STC1, there is little known function of STC1 in cancer progression. In this study, we examined the role of STC1 gene expression in human cervical cancer. We found that STC1 was down-regulated in Clinical tissues of cervical cancer. Subsequently, we found that STC1 low expression promoted cell growth, migration and invasion. We also found that STC1 overexpression inhibited cell proliferation and invasion of cervical cancer cells. Moreover, STC1 overexpression sensitized CaSki cells to drugs. These data supported the pro-apoptotic function of STC1. Further, we showed that NF-κB p65 protein directly bound to STC1 promoter and activated the expression of STC1 in cervical cancer cells.

## Results

### STC1 was Down-regulated in Clinical Tissues of Cervical Cancer

**Figure 1:**
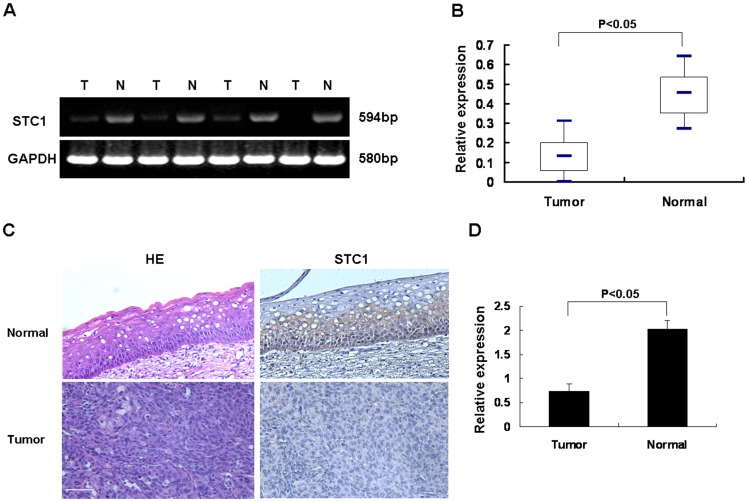
The expression of STC1 was down-regulated in cervical cancer tissues (15 cases). (A) The expression level of STC1 mRNA in cancerous (T) and adjacent normal (N) matched cervical tissues was examined by RT-PCR. GAPDH served as a loading control. (B) Box plot graph showed the quantitative results of STC1 mRNA expression in all pairs of samples (*p*<0.05). (C) Protein level of STC1 in tumor and normal tissues were examined by immunohistochemistry analysis. Bar size: 100 µm. (D) Quantity analysis of protein level of STC1 expression. The intensity of reactivity was scored using a three-tier system (*p*<0.05). Data was expressed as mean ± SEM of three separated experiments. A value of P<0.05 was considered as statistical significance.

To explore the role of STC1 in cervical cancer, we first examined the mRNA expression level in 15 pairs of matched cervical tissues by RT-PCR. The expression level of STC1 mRNA in cervical cancer tissues was decreased compared with the adjacent normal ones (Figure 1A and B). Then, immunohistochemistry analysis revealed that the protein level of STC1 was low in tumor tissues, and while increased in adjacent normal tissues, indicating its potential role in the progression of cervical cancer (Figure 1C and D).

### Down-regulation of STC1 Promoted CaSki Cells Growth and Invasion

**Figure 2:**
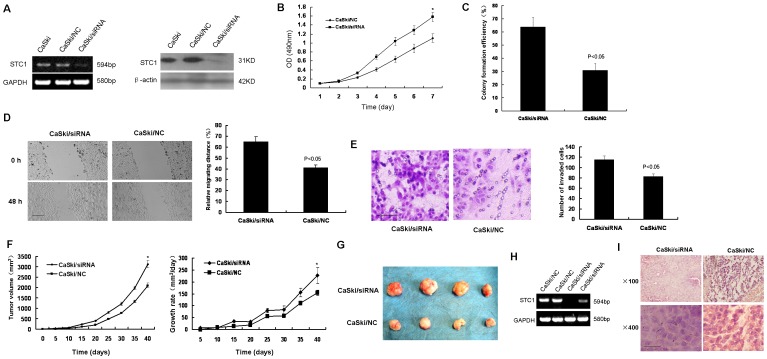
Down-regulation of STC1 promoted CaSki cells growth and invasion. (A) Knock down of STC1 in CaSki cells. CaSki cells were transfected by STC1 targeting siRNA, and knockdown efficiency was shown by RT-PCR and western blotting. (B) MTT assays showed that the effect of decreased STC1 on CaSki cell growth. Following a 7-day period, the growth of CaSki/siRNA cells was much faster than CaSki/NC cells (*p<0.05). (C) Colony formation assay demonstrated the large number of cell colonies from CaSki/siRNA cells compared to CaSki/NC cells (*p*<0.05). (D) Wound healing assays showed the effect of STC1 on the migration of CaSki cells. CaSki/siRNA cells migrated faster compared to CaSki/NC cells (left panel). The relative migration distance of CaSki cells was calculated (right panel) (*p*<0.05). Bar size: 100 µm. (E) Matrigel invasion assays showed the effect of STC1 on the invasion of CaSki cells. The number of CaSki/siRNA cells on the filter surface was larger than CaSki/NC cells (left panel) (*p*<0.05). The mean value of invaded cells was shown in right panel. Bar size: 100 µm. (F) The growth curves of the xenografts were determined by tumor volume (left panel) (*p<0.05). The growth rates of the xenografts were valuated by tumor volume/days (right panel) (*p<0.05). CaSki/siRNA or CaSki/NC cells were injected subcutaneously into nude mice. (G) At end of experimental period, the final xenograft tumors were shown. (H) RT-PCR analyzed the expression of STC1 in representative xenograft tumors. (I) Representative images of histological inspection of xenograft tumors. The sections of xenograft tumors were stained with H&E. Bar size: 20 µm. Data was expressed as mean ± SEM of three separated experiments. Data was expressed as mean ± SEM of three separated experiments. A value of P<0.05 was considered as statistical significance.

To study the biological function of STC1, we generated RNAi vector containing siRNA specifically targeting STC1 to stably knock down the endogenous expression of STC1 in CaSki cells. As shown in Figure 2A, comparing to the control (CaSki/NC), cells transfected with siRNA -STC1 had significantly decreased levels of STC1 mRNA or protein.

Subsequently, we examined the effect of decreased STC1 on CaSki cell growth by MTT assays. Following a 7-day period, the growth of CaSki/siRNA cells was much faster than CaSki/NC cells, and significantly high numbers of CaSki/siRNA cells were observed from day 4(Figure 2B). A similar pattern of promote effect of reduced STC1 expression in CaSki cells was achieved in colony formation assay (Figure 2C). Therefore, the high activity and a large number of cell colonies from CaSki/siRNA cells demonstrated that down-regulation of STC1 expression promoted cell growth in vitro.

Cell migration and invasion is important process of tumor development and metastasis. Next, we examined the effect of STC1 on the migration of CaSki cells by the wound healing assay in Figure 2D. Following incubation of physically wounded cells for 48 h, CaSki/siRNA cells migrated faster compared to CaSki/NC cells. Further analysis of the impact of STC1 on CaSki cells invasion revealed that down-regulation of STC1 enhanced the invasion of cells in vitro, determined by matrigel invasion assay (Figure 2E). The significantly longer distance of migration and large numbers of CaSki/siRNA cells on the filter surface indicated that down-regulation of STC1 enhanced the migration and invasion ability of CaSki cells in vitro.

To further determine the role of STC1 in tumorigenicity and development of cervical cancer, CaSki/siRNA or CaSki/NC cells were injected subcutaneously into nude mice. The development of tumor was monitored for 40 days. As shown in Figure 2F, STC1 knockdown tumors emerged earlier and grew rapidly compared to control tumors. At end of experimental period, the final weights of STC1 knockdown tumors (1.417±0.169 g) were found to be markedly higher than controls (0.478±0.115 g) (Figure 2G). RT-PCR of STC1 in representative xenograft tumors indicated that decreased STC1 expression had been maintained throughout experimental time course (Figure 2H). H&E staining of STC1 knockdown tumors showed a high nuclear/cytoplasmic ratio, large, and deep-stained nuclei compared to control tumors(Figure 2I). Collectively, these data evidenced that down regulation of STC1 promoted in vivo xenograft tumor development.

### Overexpression of STC1 Inhibited Cell Proliferation and Invasion of CaSki Cells

**Figure 3:**
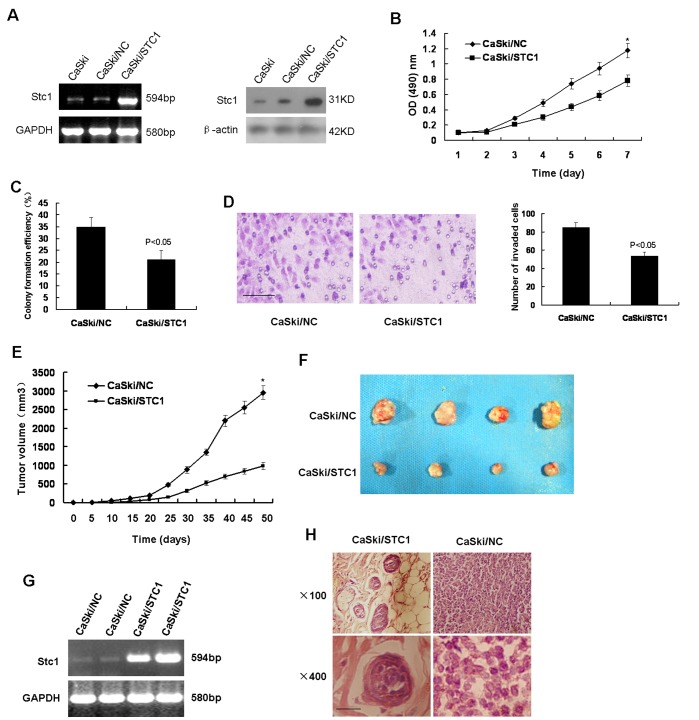
Overexpression of STC1 inhibited cell proliferation and invasion of CaSki cells. (A) Overexpression of STC1 in CaSki cells. STC1 expression vector was transfected into CaSki cells, and increased expression of STC1 was shown by RT-PCR and western blotting. (B) MTT assays showed that the growth of CaSki/STC1 cells was much slower than CaSki/NC cells (*p<0.05). (C) Colony formation assay showed that a small amount of cell colonies from CaSki/STC1 cells demonstrated a low activity (*p*<0.05). (D) Matrigel invasion assay revealed that up-regulation of STC1 mitigated the invasion of cells in vitro. Bar size: 100 µm. (E) STC1 overexpressed tumors emerged later and slowly grew compared to control tumors (*p<0.05). (F) At end of experimental period, the final weights of STC1 overexpressed tumors were found to be lower than controls. (G) RT-PCR of STC1 in xenograft tumors indicated that increased STC1 expression had been maintained throughout experimental time course. (H) H&E staining of STC1 overexpressed tumors showed a low nuclear/cytoplasmic ratio, and limited to the cancer nests compared to control tumors. Bar size: 20 µm. Data was expressed as mean ± SEM of three separated experiments. A value of P<0.05 was considered as statistical significance.

Given that down-regulation of STC1 promoted CaSki cells development in vitro or in vivo, we hypothesized that STC1 may inhibited CaSki cells development. To test this hypothesis, CaSki cells were transfected with plasmid encoding STC1. Comparing to the control (CaSki/NC), cells (CaSki/STC1) transfected with plasmid encoding STC1 had increased levels of STC1 mRNA or protein (Figure 3A). MTT assays showed that the growth of CaSki/STC1 cells was much slower than CaSki/NC cells (Figure 3B). Colony formation assay showed that a small amount of cell colonies from CaSki/STC1 cells demonstrated a low activity (Figure 3C). Matrigel invasion assay revealed that up-regulation of STC1 mitigated the invasion of cells in vitro (Figure 3D).

Next, CaSki/STC1 or CaSki/NC cells were injected subcutaneously into nude mice. STC1 overexpressed tumors emerged later and grew slowly compared to control tumors (Figure 3E). At end of experimental period, the final weights of STC1 overexpressed tumors (0.285±0.057 g) were lower than controls (0.523±0.154 g) (Figure 3F). RT-PCR of STC1 in xenograft tumors indicated that increased STC1 expression had been maintained throughout experimental time course (Figure 3G). H&E staining of STC1 overexpressed tumors showed a low nuclear/cytoplasmic ratio, and limited to the cancer nests compared to control tumors(Figure 3H). Collectively, these data suggested that up-regulation of STC1 inhibited cell proliferation and invasion of CaSki cells in vitro or in vivo.

### STC1 Sensitized CaSki Cells to Drugs

**Figure 4:**
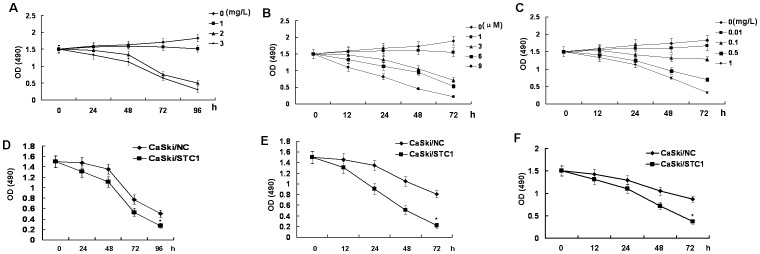
STC1 sensitized CaSki cells to drugs. (A) Effect of cisplatin on CaSki cells growth. CaSki cells were treated with with or without cisplatin (0, 1, 2, and 3 mg/L) for 96 h, removing aliquots every 24 h to evaluate cell viability. (B) Effect of thapsigargin on CaSki cells growth. CaSki cells were treated with with or without thapsigargin (0, 1, 3, 6, and 9 µM) for 72 h, removing aliquots every 24 h to evaluate cell viability. (C) Effect of rapamycin on CaSki cells growth. CaSki cells were treated with with or without rapamycin (0, 0.01, 0.1, 0.5, and 1 mg/L) for 72 h, removing aliquots every 24 h to evaluate cell viability. (D) STC1 sensitized CaSki cells to cisplatin. CaSki/STC1 or CaSki/NC cells were treated with cisplatin (2 mg/L) for 96 h. MTT assays detected the cell growth of CaSki/STC1 or CaSki/NC cells in the face of cisplatin (*p<0.05). (E) STC1 sensitized CaSki cells to thapsigargin. CaSki/STC1 or CaSki/NC cells were treated with thapsigargin (3 µM) for 72 h. MTT assays detected the cell growth of CaSki/STC1 or CaSki/NC cells in the face of thapsigargin (*p<0.05). (F) STC1 sensitized CaSki cells to rapamycin. CaSki/STC1 or CaSki/NC cells were treated with rapamycin (0.5 mg/L) for 72 h. MTT assays detected the cell growth of CaSki/STC1 or CaSki/NC cells in the face of rapamycin (*p<0.05). Data was expressed as mean ± SEM of three separated experiments. A value of P<0.05 was considered as statistical significance.

Previous results showed that STC1 may inhibit cell proliferation of CaSki cells. Cisplatin, thapsigargin and rapamycin are drugs associated with cells growth and apoptosis. Cisplatin-based combination chemotherapy has commonly been used in tumor therapy including cervical cancer [23]. Thapsigargin as an apoptotic agent can exhibit effectively tumor control based on the ability to inhibit the intracellular sarco−/endoplasmtic calcium pump [24], [25]. The mammalian target of rapamycin is a serine/threonine protein kinase of the PI3K/AKT signaling pathway which plays a critical role in controlling cancer cellular growth, metabolism and cell cycle progression [26]. Next, CaSki cells were treated with with or without cisplatin (0, 1, 2, and 3 mg/L), thapsigargin (0, 1, 3, 6, and 9 µM), or rapamycin (0, 0.01, 0.1, 0.5, and 1 mg/L) for 96 h or 72 h, removing aliquots every 24 h to evaluate cell viability. We found that the growth of CaSki cells was slow with the enhancement of time and concentration of drugs (Figure 4A–C). To determine if expression of STC1 increased drug-induced cells growth retard, CaSki/STC1 or CaSki/NC were treated with different drugs such as cisplatin, thapsigargin and rapamycin. The growth of CaSki/STC1 cells was slower with increasing of time compared to CaSki/NC cells when cells were treated with cisplatin, thapsigargin or rapamycin (Fig. 4D–F). These data demonstrated that overexpression of STC1 enhanced the sensitivity of CaSki cells to drugs.

### Direct Binding of NF-κB p65 Protein to STC1 Promoter Region in Cervical Cancer Cells and Regulated the Expression of STC1

**Figure 5:**
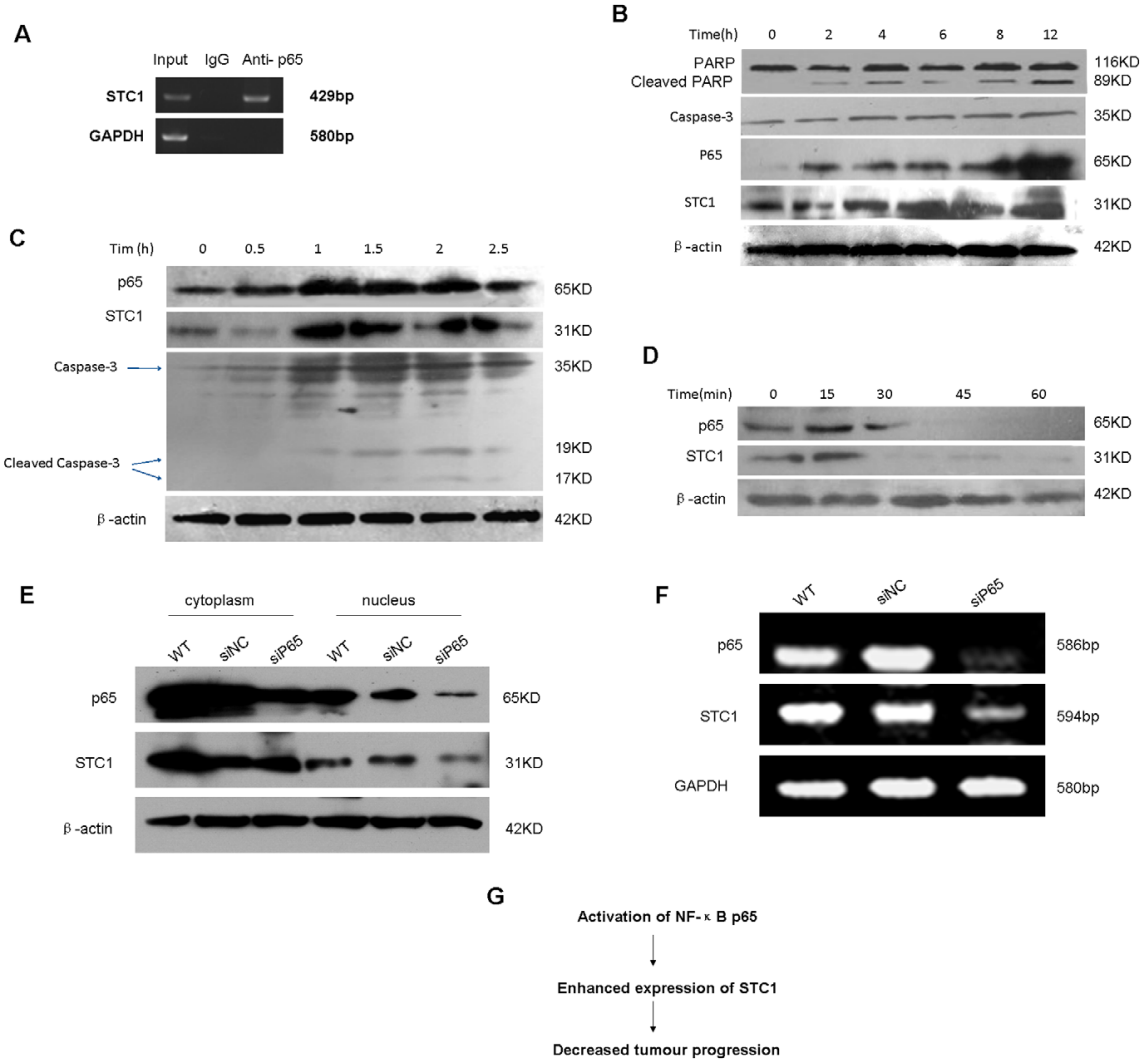
Direct binding of NF-κB p65 protein to STC1 promoter and regulated the expression of STC1. (A) The binding sites of STC1 were tested in CaSki cells by chromatin coimmunoprecipitation. The site was found to bind to NFκB p65. (B) Western blotting detected the activity of PARP, caspase-3, STC1, and NF-κB p65 in CaSki cells. CaSki cells were treated with thapsigargin (3 µM) for 12 h. (C) Western blotting detected the activity of p65, STC1, and caspase-3 in CaSki cells. CaSki cells were treated with increasing time of TNFα (10 mg/L) for 2.5 h. (D) Western blotting detected the activity of p65 and STC1 in CaSki cells. CaSki cells were treated with PDTC (10 µM) for 60 min. (E) Subcellular activity of p65 and STC1 in CaSki cells was analyzed by Western blotting. CaSki cells were treated with siRNA knockdown of p65 for 72 h. (F) The expression of p65 and STC1 in CaSki was detected by RT-PCR at mRNA level. CaSki cells were treated with siRNA knockdown of p65 for 48 h. (G) Schematic representation of some findings in this work.

The nuclear factor-kappa B (NF-κB) family of transcription factors regulates the expression of a lot of genes including proliferation and invasion genes. NF-κB pathway plays a pivotal role in cancers and cardiovascular disease. The promoter of STC1 contains multiple NF-κB p65 binding sites [27]. To begin further exploring mechanisms between the correlation of STC1 and p65, the binding sites were tested in CaSki cells by chromatin coimmunoprecipitation. The site (-GGGACAAACC-) was found to bind to NF-κB p65 (Fig. 5A). To determine if STC1 expression in CaSki cells was correlated with NF-κB, the activity of NF-κB was detected. Accompanying with enhanced activity of PARP and Caspase-3, the activity of NF-κB p65 and STC1 also increased when CaSki cells were treated with thapsigargin (Fig. 5B). TNFα activates apoptotic pathways following simultaneous induction of NF-κB. To determine if expression of STC1 similarly increased in TNFα-induced apoptosis, CaSki cells were treated with increasing time of TNFα. The expression of NF-κB p65 increased and the activity of STC1 and Caspase-3 enhanced in response to TNFα (Fig. 5C). Pyrrolidine dithiocarbamate (PDTC) is an effective inhibitor of NF-κB activation and suppresses NF-κB dependent transcription of pro-inﬂammatory cytokines and chemokines [28]. When CaSki cells were treated with PDTC, the activity of NF-κB p65 decreased and the expression of STC1 down-regulated with increasing time of PDTC (Fig. 5D). Furthermore, after the knockdown of p65 was performed by transfecting siRNA targeting NF-κB p65, the subcellular expression of p65 and STC1 in CaSki cells was analyzed (Figure 5E). The expression of p65 in cytoplasm and nucleus both decreased, and while the one in nucleus significantly reduced. The down-regulating STC1 expression in nucleus was also found following the knockdown of p65. The knockdown of p65 caused the expression of p65 and STC1 both decreasing at mRNA level (Figure 5F).

## Discussion

There is mounting evidence that STC1 is tightly associated with the development of cancer. But the expression of STC1 varied in different tissues and even for the same tissue in different level (mRNA or protein), and therefore the function of STC1 might show the difference [29]. In this study, we found that STC1 inhibited cell proliferation and invasion of cervical cancer cells. STC1 is down-regulated in Clinical tissues of cervical cancer, which indicated STC1 is involved in the progression of cervical cancer. In cervical cancer, the low expression of STC1 might cause tumor development. Subsequently, we found that STC1 low expression promoted cell growth, migration and invasion after the down-regulation of STC1 by RNA interference in cervical cancer cells. Further, we also found that STC1 overexpression inhibited cell proliferation and invasion of cervical cancer cells. Moreover, STC1 overexpression sensitized CaSki cells to drugs. These data support the option that STC1 may inhibit tumor progression for cervical cancer. The loss of function of STC1, which inhibit the growth and invasion of tumor cells, might lead to tumor progression in cervical cancer.

The promoter of STC1 gene contains HIF and NF-κB binding site. It is reported that HIF-1α bound to STC1 gene promoter and p300 was required for a productive interaction with HIF-1 and the upregulation of STC1 mRNA and protein expression [30]. Chen et al thought that STC1 partially blocked NF-κB activation in TNF-α–treated human coronary artery endothelial cells [31]. Law et al reported that histone hyper-acetylation and the recruitment of activated NFκB stimulated STC1 gene expression in HT29 cells [22]. The relationship between STC1 and NF-κB is different in different tissues type. We showed that NF-κB p65 protein directly bound to STC1 promoter and regulated the expression of STC1 in cervical cancer cells. These results have potential clinical implications. Our results suggest that enhancing the expression of STC1 should be a good strategy to inhibit tumor proliferation and invasion and also might be a feasible combined treatment with chemotherapeutic drugs in cervical cancer.

From above these results, we put forward the model for STC1-mediated inhibiting of tumor progression (Figure 5): After activation of the NF-κB p65, STC1 expression increased. The elevated STC1 expression decreased tumor progression, and accordingly the sensitivity of the cervical cancer cells with elevated STC1 expression to the chemotherapy drug increased. Thus, further studies are to elucidate deeply the mechanisms involved in STC1-mediated inhibiting of tumor progression and to further explore what other molecules take part in the progression.

## Materials and Methods

### Samples and Immunohistochemistry

Cases of cervical cancer and adjacent normal tissues were obtained on protocols approved by the Secondary Xiangya Hospital of Central South University. Clinicopathologic characteristics of the 15 patients with cervical cancer were showed in Table S1. Paraffin sections were deparaffinized with xylene and rehydrated in graded alcohol. Endogenous peroxidase activity was blocked by incubating in 3% hydrogen peroxide at RT for 10 min. Non-specific binding was blocked with PBST containing 10% goat serum for 2 h at RT. STC1 antibody (Santa Cruz Biotechnology) was added to each slide and incubated at 4°C overnight. Following three washes, slides were incubated with Envision (DAKO) for 40 min at RT. Diaminobenzidine was used as a chromogen. Sections were counterstained with hematoxylin, dehydrated, and mounted. Evaluation of immunohistochemical slides was done with a Nikon Eclipse E800 microscope at 200× magnification. The intensity of reactivity was scored using a three-tier system: 0–3(0 = no staining, 3 = 100% staining). The immunoreactive score of the sample was determined by the percentage of positive cells. The study protocol was approved by the Ethics Committee of Xiangya School of Medicine, Central South University.

### Reverse Transcription-PCR (RT-PCR)

RNA isolated from tissues and cells was reversely transcribed and amplified using the ONE-STEP reverse transcription-PCR System (Fermentas). Primer sequences used were shown in Table S2. After heating at 95°C for 1 min, PCRs were exposed to 30 cycles (GAPDH, 25 cycles) of 95°C for 30 s, 60°C for 30 s, and 68°C for 1 min 30 s with a final extension at 68°C for 10 min. The integrated density value (IDV) of each band was assessed using Gel-Pro Image Analyzer (Bio-Rad, Hercules, Calif), and the ratios of IDV values were calculated to evaluate the relative expression levels of the mRNA.

### Cell Culture

CaSki human cervical cancer cells were maintained by our lab and cultured in Dulbecco-modified Eagle medium (DMEM; Gibco) supplemented with 10% bovine calf serum (BCS) (Gibco). Cells were maintained at 37°C in an atmosphere of humidified air with 5% CO2.

### Vector Construction and Cell Transfection

To knock down STC1 expression, we used pRNAT-U6.1/Neo vector encoding a small hairpin RNA directed against the target gene in CaSki cells. The target sequences for STC1 were 5′- TTAGTCCAGGAAGCAATAGTA -3′ (CaSki/siRNA). We used negative universal control as a negative control (CaSki/NC).

For transfection of the plasmid expression vector encoding human STC1 the DNA sequencing containing the STC1 open reading frame flanked by HindIII-XhoI restriction sites was PCR amplified from CaSki cells. Primer sequences used were sense 5′- GAAAGCTTATGCTCCAAAACTCAG -3′ and antisense 5′- TTCTCGAGTTATGCACTCTC ATGG -3′. The resulting fragment was inserted into HindIII/XhoI -precut pcDNA3.1 (+) (Invitrogen) to generate pcDNA3.1- STC1. The desired sequence was confirmed by direct DNA sequencing.

For transfection, cervical cancer cells were grown to 70% confluence and transfected in serum-free medium for 6 h with lipofectamine 2000 (Invitrogen) and vector. After 48 h, cells were harvested, followed by limited dilution in 96-well plates for the generation of individual cell clones. Three weeks later, the levels of STC1 expression in cell clones that had been infected with vectors, were characterized for the STC1 protein and mRNA.

### MTT and Colony Formation

MTT assay was performed as reported previously [32]. For colony formation assay, cells at 100 cells per well in 60 mm plates were cultured in 10% FBS DMEM at 37°C 5% CO2 for 3 weeks. The cell colonies were washed twice with PBS, fixed by 4% paraformaldehyde for 15 min and stained with Gimsa for 30 min. Individual clones with more than 50 cells were counted. Clone forming efficiency for individual type of cells was calculated, according to the number of colonies/number of inoculated cells ×100%.

### Monolayer Wound Healing and Invasion Assays

For monolayer wound healing assay, cells were grown in 10% FBS DMEM in 60 mm plates. After the cells reached sub-confluence, the monolayer cells were wounded by scraping off the cells and then grown in medium for 48 h. The migrated distance of CaSki cells was monitored and imaged under a microscope. The distances of cell migration were calculated by subtracting the distance between the lesion edges at 48 h from the distance measured at 0 h. The relative migrating distance of cells is measured by the distance of cell migration/the distance measured at 0 h. For invasion assay, cells at 10^4^/well were seeded in the upper chambers in 200 µL FBS-free DMEM and the lower wells was filled with 500 µL 10% FBS DMEM for inducing cell migration. Following incubation for 24 h, the cells on the filter surface were fixed with 4% formaldehyde, stained with 0.5% crystal violet, and examined under a microscope. Cells in at least six random microscopic fields (200×) were counted.

### Western Blot Analysis

The cells were washed with cold phosphate-buffered saline (PBS) and lysed in Laemmli buffer (62.5 mM Tris–HCl, pH 6.8, 2% SDS, 10% glycerol, 50 mM dithiothreitol, and 0.01% bromphenol blue) for 5 min at 95°C. Cell lysates were analyzed by SDS/PAGE and transferred electrophoretically to polyvinylidene difluoride membrane. The blots were probed with specific antibodies by a secondary detection step. The immunoreactive proteins were revealed by an ECL kit. Western blot was carried out using the following antibodies: Rabbit anti-STC1 antibody (Santa Cruz Biotechnology), Rabbit anti-Phospho-NF-κB p65 antibody (Cell Signaling Technology), Rabbit anti-PARP antibody (Cell Signaling Technology), Rabbit anti-Caspase-3 antibody (Cell Signaling Technology).

### In Vivo Tumor Growth Assay

The influence of STC1 on the tumor development of cervical carcinoma in vivo was examined. Cells at 5×10^6^ per mouse were injected subcutaneously into 4 weeks old Balb/c nude mice (n = 4 per group, Shanghai Laboratory Animal Center, Shanghai, China). The experimental pairs were done in different mice. The development and growth of solid tumors were monitored by measuring tumor size using a vernier caliper in a blinded fashion every five days for a 40-days period. The tumor volume was calculated using the formula: tumor volume (mm^3^) = width (mm)^ 2^×length (mm) ×0.5 [33]. At the end of the experiment, all mice were sacrificed and individual tumor weights were measured using a balancer.

### Chromatin Immunoprecipitation Assays

ChIP assay was conducted using the ChIP assay kit according to the manufacturer’s instruction (Upstate Biotechnology, Lake Placid, NY) and antibody against NF-κB p65 (Cell Signaling Technology, Beverly, MA,USA). Chromatin immunoprecipitation primers were designed to amplify a fragment containing STC1 promoter. The primer sequences used were shown in Table S2.

### Statistical Analysis

Data were expressed as mean ± SEM. The difference among groups was determined by ANOVA analysis and comparison between two groups was analyzed by the Student’s t-test using the GraphPad Prism software version 4.0 (GraphPad Software, Inc., San Diego, CA). A value of P<0.05 was considered as statistical significance.

## Supporting Information

Table S1 **Clinicopathologic characteristics of the 15 patients with cervical cancer.**
(DOC)

Table S2 **Primer for RT-PCR.**
(DOC)
