# Supplementary material for: Stanniocalcin1 (STC1) Inhibits Cell Proliferation and Invasion of Cervical Cancer Cells
Source: PLoS One. 2013 Jan 29;8(1):e53989. doi: 10.1371/journal.pone.0053989 (PMC3558422; doi:10.1371/journal.pone.0053989)
Supplement: Table S1 — Clinicopathologic characteristics of the 15 patients with cervical cancer. (DOC) [file pone.0053989.s001.doc]

Table S1 Clinicopathologic characteristics of the 15 patients with cervical cancer.

| Variable | Cases (n) |
| --- | --- |
| Gender |  |
| Male | 9 |
| Female | 6 |
| Age |  |
| ≤ 60 | 7 |
| >60 | 8 |
| Differentiation |  |
| Well | 2 |
| Moderately | 6 |
| Poorly | 7 |
| LN metastasis |  |
| Negative | 4 |
| Positive | 11 |
| Stage |  |
| I+II | 6 |
| III+IV | 9 |
