# Supplementary material for: Stanniocalcin1 (STC1) Inhibits Cell Proliferation and Invasion of Cervical Cancer Cells
Source: PLoS One. 2013 Jan 29;8(1):e53989. doi: 10.1371/journal.pone.0053989 (PMC3558422; doi:10.1371/journal.pone.0053989)
Supplement: Table S2 — Primer for RT-PCR. (DOC) [file pone.0053989.s002.doc]

Table S2 Primer for RT-PCR.

| **Primers Sequence 5’ – 3’** | **Gene** |
| --- | --- |
| TTCTGGTGCTGGTGATCAGTG | forward STC1 |
| TTTGGGCACAGTGGTCTGTCT | reverse STC1 |
| AATCCCATCACCATCTTCCA | forward GAPDH |
| CCTGCTTCACCACCTTCTTG | reverse GAPDH |
| GATGCAAAGTAAAGCCACTGG | forward STC1 promoter |
| CAATAAGCTGGCCAAAGCAA | reverse STC1 promoter |
